# Burnout Prevalence and Its Associated Factors among Malaysian Healthcare Workers during COVID-19 Pandemic: An Embedded Mixed-Method Study

**DOI:** 10.3390/healthcare9010090

**Published:** 2021-01-17

**Authors:** Nurhanis Syazni Roslan, Muhamad Saiful Bahri Yusoff, Ab Razak Asrenee, Karen Morgan

**Affiliations:** 1 Department of Medical Education, School of Medical Sciences, Universiti Sains Malaysia, Kelantan 16150, Malaysia; msaiful_bahri@usm.my; 2 Department of Psychiatry, School of Medical Sciences, Universiti Sains Malaysia and Hospital USM, Universiti Sains Malaysia, Kelantan 16150, Malaysia; asrenee@usm.my; 3 Perdana University-Royal College of Surgeons in Ireland School of Medicine, Kuala Lumpur 50490, Malaysia; karenmorgan@perdanauniversity.edu.my; 4 Department of Health Psychology, Royal College of Surgeons in Ireland, D02 YN77 Dublin, Ireland

**Keywords:** psychological well-being, burnout, health personnel, caregiver, pandemic, COVID-19

## Abstract

Coronavirus disease 2019 (COVID-19) has become a global health threat and has placed an extraordinary demand on healthcare workers around the world. In this study, we aim to examine the prevalence of burnout and its associated factors and experience among Malaysian healthcare workers during the COVID-19 pandemic through an embedded mixed-method study design. We found that more than half of Malaysian healthcare workers in this sample experienced burnout. Direct involvement in COVID-19 screening or treatment, having a medical condition, and less psychological support in the workplace emerged to be the significant factors in personal-, work-, and patient-related burnout. Participants described their workloads, uncertainties caused by the pandemic, challenging work–family balance, and stretched workplace relationships as the sources of burnout. Exhaustion appeared to be the major symptom, and many participants utilized problem-focused coping to deal with the adversities experienced during the pandemic. Participants reported physical-, occupational-, psychological-, and social-related negative impacts resulting from burnout. As the pandemic trajectory is yet unknown, these findings provide early insight and guidance for possible interventions.

## 1. Introduction

In late December 2019, an increasing number of patients with pneumonia of an unknown etiology were seen in Wuhan, China. The causative virus was later identified as severe acute respiratory syndrome coronavirus 2 (SARS-CoV-2), and this novel pneumonia is called coronavirus disease 2019 (COVID-19) [1]. It immediately became a global public health threat due to its strong infectivity, even in the incubation period [2]. With the rapid increase of cases reported outside China, the World Health Organization (WHO) declared the COVID-19 outbreak a pandemic on 11 March 2020 [3]. As of 16 December 2020, 73.5 million people have been infected with COVID-19, with more than 1.6 million deaths reported worldwide.

The high transmissibility of the virus without the presence of vaccines can severely stretch healthcare workers (HCW) [4]. On top of facing a higher risk of infection from patient care [5] and a lack of personal protective equipment (PPE), HCW also fear transmitting the infection to their families and struggle with guilt over their patients and family members [6]. During the previous SARS outbreak in 2003, stress was observed in 57% of a sample of HCW [7]. In the COVID-19 pandemic, studies have shown higher anxiety levels among Chinese HCW as compared to the general population [8,9,10]. About 42.5% of Thai HCW were identified to have at least mild anxiety symptoms [11]. Another study revealed that 64.7%, 51.6%, and 41.2% of Turkish HCW displayed symptoms of depression, anxiety, and stress, respectively [12].

While several studies outlined the prevalence of depression, anxiety, and stress, less is known about HCW burnout. Burnout can be defined as a syndrome resulting from chronic workplace stress that has not been successfully managed [13]. Burnout is not synonymous with fatigue, stress, or depression [14] and is known to affects helping professions such as HCW. It is characterized by energy depletion or emotional exhaustion, negativity related to one’s job, and reduced professional efficacy [15,16]. Previous research has linked burnout to various personal and patient care impacts, such as decreased professionalism, empathy, patient safety, teamwork and increased medical errors and attritions [17,18,19]. A high prevalence of burnout has been seen among HCW post-natural disasters [20,21]. We postulated that there is a similar occurrence in a pandemic setting. Burnout can result from an increased work demand and decreased job resources, along with value conflicts [22], and this link has been further magnified during COVID-19. Burnout can also emanate from disproportionately huge effort and low satisfaction, making the most dedicated HCW feel vulnerable, especially in pandemic times [16].

For the past two decades, burnout research has identified several risk factors at the individual and situational levels [23]. At the individual level, younger age has been consistently associated with a higher risk of developing burnout [18,24]. Some studies also found that single HCW were at a higher risk for burnout as compared to their married counterparts [24], while having children has been associated with a lower risk of developing burnout [25]. The role of spiritual well-being was also explored, as it influenced burnout development through emotional intelligence [26]. At the situational level, long hours have been found to be associated with burnout, with each additional hour worked per week increasing the risk of developing burnout by 2% [24,25]. In line with the Job Demand–Resource theory [27], poor health and inadequate psychological support in the workplace have been linked to burnout development [28,29], with mixed results [30]. These associations have yet to be explored in the context of a pandemic and may provide understanding that is important in designing effective burnout prevention measures for HCW.

In Malaysia, the healthcare system has increased its COVID-19 preparedness since 6 January 2020 when screening and surveillance measures were strengthened. The first case was detected on 24 January 2020, and the Restriction of Movement Order (RMO) was put into place on 18 March 2020 [17]. As of 16 December 2020, 86,618 cases have been detected, with 422 deaths reported, in the country. With the increasing trend of confirmed cases, HCW continue to serve the country in the various roles of surveillance, screening, diagnosis, and treatment. Thus, building from the above conceptual framework, we aim to examine burnout prevalence and its association with demographic characteristics among Malaysian HCW and explore their burnout experiences during the COVID-19 pandemic in an embedded mixed-method study. We hypothesized that burnout prevalence is high among HCW and factors such as direct involvement with COVID-19, long hours, being young or single, having a medical condition, having no children or having inadequate childcare support, inadequate psychological support in the workplace, and irregular spiritual routines were associated with a higher risk of developing burnout.

## 2. Materials and Methods

### 2.1. Study Design

As the phenomenon of burnout in the context of a pandemic is less understood, we adopted a mixed-method approach with an embedded design [31,32]. In order to estimate the prevalence of burnout among Malaysian HCW, we conducted a cross-sectional study using an online instrument. Through a descriptive phenomenological approach, we captured the HCW experience using open-ended questions to explore the main quantitative data from the cross-sectional study.

### 2.2. Participants

Using snowball sampling, we invited Malaysian assistant medical officers, doctors, health inspectors, hospital food preparation personnel, medical laboratory technologists, nurses, paramedics, pharmacists, physicians, physiotherapists, dieticians, therapists, psychologists, counsellors, radiographers, and social workers from public and private healthcare services to enroll in this study. The required sample size for the study, with 9 predictors, an anticipated effect size of 0.02, and 30% a nonresponse rate, was 1126 [33].

### 2.3. Instrument

The instrument package included sociodemographic questions, the Copenhagen Burnout Inventory (CBI), and open-ended questions. The demographic questions included the workplace setting, involvement with COVID-19 patients, working hours, age, marital status, number of children, availability of childcare at home, medical conditions, perceived psychological support at the workplace, and spirituality routines.

The CBI is an instrument to measure occupational burnout with excellent psychometric properties and is available in the public domain. It has three dimensions that include personal-related (6 items), work-related (7 items), and patient-related burnout (6 items). Each item was rated on a scale of always/to a very high degree (100), often/to a high degree (75), sometimes/somewhat (50), seldom/to a low degree (25), and never/to a very low degree (0) [34]. The CBI was also validated in a pandemic context, with a Cronbach’s alpha coefficient of 0.94 [35]. For this study, we used the Malay translated CBI (CBI-M), which was validated and found to have a Cronbach’s alpha coefficient of 0.83–0.87 [36]. We calculated the average scores for each dimension, where an average score of 50% or above is treated as burnout [37].

At the end of the instrument, all participants were invited to answer four optional self-declared open-ended questions. These questions were tested to ensure that they were able to capture the intended research questions. The final questions were as follows:

If you have experienced burnout, you are invited to share your experience below:

1. What caused burnout in yourself?
2. What were the burnout symptoms that you experienced?
3. How did you try to cope with the symptoms?
4. How did the symptoms affect your work or your life (if at all)?

### 2.4. Data Collection

In line with the RMO, we conducted the study using Google Forms between 21 April 2020 and 20 May 2020. During the beginning of the data collection, Malaysia was in its third month of facing COVID-19 under the second phase of RMO. We sent the invitation link through the Ministry of Health official website and HCW-related nongovernmental organizations Facebook pages. In the informed consent form, we emphasized to the participants the anonymity and confidentiality of the data. There were no monetary incentives given, but the participants received the option to gather information on their burnout scores and helplines that may be useful.

### 2.5. Data Analysis

We analyzed the quantitative component using SPSS version 24 (IBM Corp., Armonk, N.Y., USA). We applied descriptive statistics to examine the personal-, work-, and patient-related burnout prevalences. We performed simple logistic regression to assess the relationships between each independent variable (involvement with COVID-19, working hours, age, marital status, children, availability of childcare support in the pandemic, medical conditions, perceived psychological support at the workplace, and spirituality routine) with all the dimensions of burnout. With the same set of independent variables, we performed multiple logistic regression to find significant relationships between each of the independent variables and dimensions of burnout by controlling the other independent variables. We presented both crude and adjusted odds ratios, along with 95% confidence intervals (CI) and *p*-values. In order to evaluate the nonresponse bias, we also compared the prevalences of the first 50% versus the last 50% participants who enrolled in the study.

In the qualitative analysis, we included the open-ended responses from the participants who scored more than 50% in their CBI. We analyzed the data using a thematic analysis on computer-assisted software, Atlas.Ti version 8.1 (Atlas.Ti GmbH, Berlin, Germany). NSR open-coded and organized the coding into themes. MSBY reviewed the coding and themes, and their agreement was generally concordant. In order to enhance the trustworthiness of the findings, we also used data sources triangulation and code–recode procedures (an intra-rater agreement method to enhance reliability where NSR recoded the data after some time to compare with the initial coding) during the data analysis process [38].

## 3. Results

### 3.1. Participants Characteristics

A total of 933 HCW completed the online instruments, with a response rate of 82.9%. We excluded 40 entries due to incomplete quantitative responses or duplicates. The demographic characteristics of the participants are summarized in Table 1. The highest participation was from doctors, social workers, and assistant medical officers. The majority of the participants worked in government hospitals, had indirect involvement with COVID-19 patients, worked 60 h or more weekly, were aged less than 40 years old, married, had children, had adequate childcare support at home, had no medical conditions, perceived that they received adequate psychological support at work, and practiced a regular spiritual routine.

### 3.2. Prevalence of HCW Burnout in the COVID-19 Setting

The overall prevalence of personal-, work-, and patient-related burnout in this sample of Malaysian HCW was 53.8%, 39.1%, and 17.4%, respectively. There were no significant differences in any of these burnout prevalence levels between the first 50% and last 50% of the participants. Personal-related burnout was the highest among pharmacists and HCW in district health offices, work-related burnout was highest among health inspectors and HCW in medical laboratories, and patient-related burnout was highest among paramedics and HCW in private hospitals. Participants who were directly involved in screening or treating COVID-19 patients, aged less than 40 years, married, had no children, had inadequate childcare support at home, had a medical condition, perceived that they received inadequate psychosocial support at work, or had irregular spiritual routines were found to burnout more in all three dimensions of the CBI (Table 2).

### 3.3. Demographic Associations with Burnout

Significant demographic associations with personal-, work-, and patient-related burnout are shown in Table 3 and Table 4. At the univariate level, we found that inadequate self-perceived psychosocial support at work, long hours, direct involvement with COVID-19, inadequate childcare support at home, medical conditions, younger age, being single, having no children, and irregular spirituality routines were significantly associated with all dimensions of burnout. At the multivariate level, younger age, direct involvement with COVID-19 patients, long hours, medical conditions, inadequate self-perceived psychological support at work, and inadequate childcare support were significantly associated with personal-related burnout. Direct involvement with COVID-19 patients, long hours, medical conditions, adequate self-perceived psychological support at work, inadequate childcare support, and irregular spirituality routines were significantly associated with work-related burnout. Younger age, direct involvement with COVID-19 patients, medical conditions, and inadequate self-perceived psychological support at work were significantly associated with patient-related burnout.

### 3.4. Qualitative Findings

#### 3.4.1. Sources of Burnout

In total, 72.7% of HCW with a high burnout score responded to the open-ended questions. Through a thematic analysis, we found that the most-described sources of burnout were their workload (long hours, working with extra precaution measures, team dynamic disruptions, the diffuse impact of pandemic, and bureaucracy matters). Some participants also described uncertainties caused by the pandemic (unpredictability of the course of the pandemic, frequent standard operating procedure (SOP) and role changes, quarantine, and disruption in their career plan); a challenged work–family balance (disruption of the work–family balance, confinement impact on the family, fear of transmitting COVID-19 to family, and financial loss); and stretched workplace relationships (superiors, colleagues, and patients) as contributors to their burnout (Table 5).

#### 3.4.2. Burnout Symptoms

The most reported symptoms were overwhelming exhaustion (emotional, physical, and frustration). HCW also described symptoms of cynicism (distant attitude towards work and making callous comments to patients) and reduced professional accomplishment (loss of enthusiasm, a feeling of underperforming, and low self-esteem) (Table 6).

#### 3.4.3. Coping with Burnout

The most described coping mechanisms among HCW were problem-focused coping (active and planning), followed by positive thinking coping (positive reinterpretation, acceptance, and humor). Participants also utilized support-seeking to cope, including emotional, instrumental, and spiritual support. The least-mentioned coping methods were maladaptive coping, which were self-distraction, behavioral disengagement, venting, and substance abuse (Table 7).

#### 3.4.4. Impact

The impacts of burnout can be generally categorized as occupational, physical, psychological, and social (Table 8). The most-described impacts were physical (headaches, muscular pain, sleep disturbance, ailments, palpitations, appetite loss, and near accidents). Participants also reported occupational impacts, such as a lack of focus, loss of enthusiasm, and low productivity. The described psychological impacts included irritability, anger outbursts, and anxiety symptoms, while the social impacts included negatively affected family relationships and quality of life. Some participants observed no impacts from burnout in any of these categories.

## 4. Discussions

To the best of our knowledge, this is among the few studies that have looked at the impact of the COVID-19 pandemic on HCW from a mixed-method perspective. Our quantitative findings indicated that more than half of the Malaysian HCW in this sample experienced burnout. These findings was similar to studies done on Singapore HCW (49.2%) and Indian HCW (44.6%) [39,40] and were lower than the prevalence obtained from a sample of United Kingdom HCW (79%) [41]. Through use of the Maslach Burnout Inventory, other studies revealed similar prevalences—Spanish HCW (15–82%), Romanian residents (76%), Italian HCW (25–53%), Wuhan HCW (13–61%), and Japanese HCW (31.4%) [42,43,44,45,46]. While studies comparing burnout occurrences among the types of HCW and workplace settings are limited, we found that health inspectors and HCW in medical laboratories had the highest prevalences of work-related burnout. On top of their regular duties, health inspectors are heavily involved in COVID-19 contact tracking, screening, and decontamination processes [47]. HCW in medical laboratories have experienced increased workloads as Malaysia has upgraded its COVID-19 diagnostic capacity by 86% [48]. A study in Japan revealed that laboratory medical personnel were 6.1 times more likely to develop burnout when compared to physicians during COVID-19 [46].

Our findings corroborate a previous study in China, which revealed higher anxiety in HCW who were directly involved with COVID-19 [49]. In contrast, studies conducted among Wuhan and Romanian HCW showed lower burnout occurrences in HCW working on the front lines [42,44]. Consistent with other studies, HCW working more than 60 h per week were shown to have higher burnout scores. Longer hours are associated with prolonged contact, the prolonged wearing of PPE, and sleep deprivation, and this may emphasize burnout development [9].

In terms of personal demographics, younger HCW were at a higher risk of developing burnout, and similar findings were found in studies conducted in the United Kingdom, Spain, Turkey, and Japan [12,41,43,46]. This is consistent with previous research on burnout dynamics, which suggested that organizational newcomers are more prone to developing burnout when work demands outpace their resources to cope [50]. Married HCW and those with children were associated with decreased odds of developing burnout, and this is consistent with studies measuring depression, anxiety, and stress in HCW during the COVID-19 pandemic [12,51]. Families may function as social support for HCW, and strong social support was linked to a lower risk of burnout [52]. Although it was not described in previous studies, we found that inadequate childcare support during COVID-19 increased the odds of developing burnout. Long hours and unpredictable schedules can further stretch HCW during the pandemic when they cannot find childcare support [53].

Through a multivariate regression analysis, we found that having a medical condition and inadequately perceived psychological support at the workplace increased the odds of developing burnout. A medical condition might also predispose HCW more to exhaustion as compared to healthy HCW. While a study in Turkey found no association between having a medical condition and psychological distress during the pandemic [12], a study in United Kingdom HCW revealed a significant association between background illness and burnout [41]. HCW who receive adequate psychological support at work have an outlet to discuss their experiences, concerns, and emotions, and various studies have shown an association with HCW’s psychological well-being during COVID-19 [4,9,12,41].

Spiritual intelligence was found to positively impact work performances among Malaysian nurses and deemed as an important quality to cope with stressful work demands [54]. While the relationship between spiritual practices and HCW burnout in a pandemic context has not yet been established, our data support previous findings where spiritual practices were found to correlate negatively with burnout [55]. Spiritual practices provide a source of comfort and hope during adversities and are increasingly recognized as an important resource in addressing burnout [56].

Our qualitative findings can be categorized into sources of burnout, burnout symptoms, coping strategies, and impacts. While sources of burnout among HCW are less described in the literature, we found that the themes (workload, uncertainty from the pandemic, a challenged work–family balance, and stretched work relationships) were partially similar and also unique when compared to the stressors experienced in general employee contexts (safety, infobesity, quarantine, stigma, and job insecurity) [57]. Compared to the general public, who were mostly in quarantine, HCW struggled with their workloads and work–family balance as they were providing patient care. HCW also faced additional strain, as they had to wear PPE and deal with the fear of transmitting the virus to their family when they returned home. As for the symptoms, in line with the long trajectory of COVID-19, our findings are in accord with previous studies that showed exhaustion as the major symptom experienced by burnt-out HCW, followed by cynicism and a reduced feeling of personal accomplishment [42,43].

Despite the challenges faced in pandemic times, many HCW experiencing burnout described problem-focused, positive thinking, and support-seeking as methods for coping compared to maladaptive coping. This parallels a study in New York, which found that most HCW engaged in physical activities, support groups, and spiritual practices as part of their stress-reduction activities [58]. Positive thinking or optimism was found to reduce stress and exhaustion among HCW in Turkey during COVID-19 [59]. However, similar to a study by Chor et al. (2020), some HCW resorted to maladaptive coping; coping skills intervention may play some role in addressing burnout among HCW [60]. Many HCW described physical impacts, followed by occupational, psychological, and social impacts, and this seems to be consistent with a study in Italy that reported irritability, appetite change, sleep disturbance, muscle tension, and exaggerated reactions as the major perceived impacts among its HCW [45]. These findings strongly indicate that “well-being support” for HCW during the pandemic is critical and vital to ensure their well-being—well HCW, well society!

Our study has a number of limitations. First, the sample size was relatively small compared to the actual HCW population in Malaysia. However, we found no significant prevalence differences between the first 50% and last 50% participants scores, which could suggest low nonresponse bias in the sample [61]. Second, the nonprobability sampling used in this study limited its generalizability to a larger context. Third, the findings came from a cross-sectional and qualitative study design and were not causal effects. Fourth, as the burnout and sociodemographic variables were captured from a self-reported instrument, so there might be a possibility of reporting bias. Despite the limitations, these findings offer an early insight on HCW burnout in Malaysian HCW and some guidance for possible interventions.

## 5. Conclusions

The study indicates that more than half of Malaysian HCW in this sample experienced burnout during the COVID-19 pandemic. HCW with direct involvement in COVID-19, a medical condition, or inadequate psychological support at work were at higher risks of developing personal-, work-, and patient-related burnout. Through a qualitative analysis, exhaustion appeared to be the most-described symptom, and many participants described problem-focused coping to deal with their ongoing stressors. However, COVID-19 has been ongoing for a year, and continuous strain may exhaust HCW’s resources for coping. As the pandemic trajectory is yet unknown, ongoing well-being measures, support, and longitudinal studies on burnout intervention for HCW are desirable.

## Figures and Tables

**Table 1:** Demographic characteristics of the healthcare workers (HCW) enrolled in the study (*n* = 893).

Demographic Characteristics	*n* (%)
Healthcare worker categories	
Doctor	203 (22.7)
Social worker	128 (14.3)
Assistant medical officer	120 (13.4)
Medical laboratory technologist	99 (11.1)
Hospital food preparation personnel	88 (9.9)
Physiotherapist/Dietician/Therapist	69 (7.7)
Nurse	47 (5.3)
Radiographer	41 (4.6)
Pharmacist	40 (4.5)
Paramedic	25 (2.8)
Health inspector/Public health assistant	22 (2.5)
Psychologist/Counsellor	11 (1.2)
Workplace setting	
Government hospitals	607 (68.0)
Health clinics	134 (15.0)
District health offices	52 (5.8)
Medical laboratories	39 (4.4)
Private hospitals	35 (3.9)
Private clinics	26 (2.9)
Involvement with COVID 19 pandemic	
Direct (Treating/Screening)	406 (45.5)
Indirect	487 (54.5)
Working hours in COVID 19 pandemic	
Less than 60 h per week	257 (28.8)
60 h per week or more	636 (71.2)
Age	
Less than 40 years	682 (76.4)
40 years and above	211 (23.6)
Marital status	
Single	274 (30.7)
Married	619 (69.3)
No of children	
No	337 (37.7)
Yes	556 (62.3)
Childcare support at home during COVID 19 pandemic	
Had child at home with adequate support	402 (45.0)
Had child at home with inadequate support	152 (17.0)
Medical condition	
No	708 (79.3)
Yes	185 (20.7)
Perceived psychosocial support at the workplace	
Adequate	622 (69.7)
Inadequate	271 (30.3)
Spirituality routines	
Regular	757 (84.8)
Irregular	136 (15.2)

**Table 2:** Prevalence of burnout during the coronavirus disease 2019 (COVID-19) pandemic based on participants’ characteristics (*n* = 893).

Demographic Characteristics	Personal- Related Burnout *n* (%)	Work- Related Burnout *n* (%)	Patient- Related Burnout *n* (%)
Healthcare worker categories			
Overall	480 (53.8)	349 (39.1)	155 (17.4)
Doctor	129 (63.5)	104 (51.2)	54 (26.6)
Social worker	44 (34.4)	22 (17.2)	10 (7.8)
Assistant medical officer	74 (61.7)	51 (42.5)	32 (26.7)
Medical laboratory technologist	65 (65.7)	53 (53.5)	15 (15.2)
Hospital food preparation personnel	34 (39.1)	17 (19.5)	2 (2.3)
Physiotherapist/Dietician/Therapist	27 (39.1)	18 (26.1)	8 (11.6)
Nurse	26 (55.3)	21 (44.7)	7 (14.9)
Radiographer	23 (56.1)	17 (41.5)	8 (19.5)
Pharmacist	31 (77.5)	21 (52.5)	8 (20.0)
Paramedic	10 (40.0)	9 (36.0)	7 (28.0)
Health inspector/Public health assistant	14 (63.6)	14 (63.6)	3 (13.6)
Psychologist/Counsellor	3 (27.3)	2 (18.2)	1 (9.1)
Workplace setting			
Government hospitals	318 (52.4)	230 (37.9)	105 (17.3)
Health clinics	73 (54.5)	49 (36.6)	25 (18.7)
District health offices	33 (63.5)	26 (50.0)	9 (17.3)
Medical laboratories	22 (56.4)	20 (51.3)	3 (7.7)
Private hospitals	13 (50.0)	9 (34.6)	6 (23.1)
Private clinics	21 (60.0)	15 (42.9)	7 (20.0)
Involvement with COVID 19 pandemic			
Direct (Treating/Screening)	255 (62.8)	197 (48.5)	100 (24.6)
Indirect	225 (46.2)	152 (31.2)	55 (11.3)
Working hours in COVID 19 pandemic			
Less than 60 h per week	176 (68.5)	151 (58.8)	65 (25.3)
60 h per week or more	304 (47.8)	298 (31.1)	90 (14.2)
Age			
Less than 40 years	396 (58.1)	292 (42.8)	138 (20.2)
40 years and above	84 (39.8)	57 (27.0)	17 (8.1)
Marital status			
Single	160 (58.4)	127 (46.4)	61 (22.3)
Married	320 (51.7)	222 (35.9)	94 (15.2)
No of children			
No	201 (59.6)	155 (46.0)	76 (22.6)
Yes	279 (50.2)	194 (34.9)	79 (14.2)
Childcare support at home during COVID 19 pandemic			
Had child at home (adequate support)	172 (42.8)	109 (27.1)	42 (10.4)
Had child at home (inadequate support)	107 (70.4)	85 (55.9)	37 (24.3)
Medical condition			
No	351 (49.6)	252 (35.6)	110 (15.5)
Yes	129 (69.7)	97 (52.4)	45 (24.3)
Perceived psychosocial support at the workplace			
Adequate	256 (41.2)	158 (25.4)	64 (10.3)
Inadequate	224 (82.7)	191 (70.5)	91 (33.6)
Spirituality routine			
Regular	394 (52.0)	276 (36.5)	122 (16.1)
Irregular	86 (63.2)	73 (53.7)	33 (24.3)

**Table 3:** Significant associations between the demographic characteristics and burnout using simple logistic regression (*n* = 893).

Burnout Dimension and Demographic Characteristics	Crude Odds Ratio *(95% CI)	*p*-Values
Personal-related burnout		
Perceived psychosocial support received at work: Inadequate	6.81 (4.79–9.70)	<0.001
Work more than 60 h per week	2.37 (1.75–3.22)	<0.001
Child support at home: Inadequate	2.35 (1.61–3.42)	<0.001
Suffering from some medical illness	2.34 (1.66–3.31)	<0.001
Age: Less than 40 years old	2.09 (1.53–2.87)	<0.001
Direct involvement with COVID-19	1.97 (1.50–2.57)	<0.001
Spirituality routines: Irregular	1.59 (1.09–2.31)	0.017
Children: None	1.47 (1.12–1.93)	0.006
Relationship status: Single	1.31 (0.98–1.75)	0.064
Work-related burnout		
Perceived psychosocial support received at work: Inadequate	7.01 (5.11–9.63)	<0.001
Work more than 60 h per week	3.15 (2.34–4.25)	<0.001
Child support at home: Inadequate	2.29 (1.61–3.27)	<0.001
Direct involvement with COVID-19	2.08 (1.58–2.73)	<0.001
Age: Less than 40 years old	2.02 (1.44–2.84)	<0.001
Spirituality routines: Irregular	2.02 (1.40–2.92)	<0.001
Suffering from some medical illness	2.00 (1.44–2.77)	<0.001
Children: None	1.59 (1.21–2.10)	0.001
Relationship status: Single	1.55 (1.16–2.06)	0.003
Patient-related burnout		
Perceived psychosocial support received at work: Inadequate	4.41 (3.07–6.33)	<0.001
Age: Less than 40 years old	2.90 (1.70–4.92)	<0.001
Direct involvement with COVID-19	2.57 (1.79–3.68)	<0.001
Work more than 60 h per week	2.05 (1.43–2.94)	<0.001
Children: None	1.76 (1.24–2.49)	0.002
Suffering from some medical illness	1.75 (1.18–2.59)	0.005
Child support at home: Inadequate	1.70 (1.12–2.58)	0.013
Spirituality routines: Irregular	1.67 (1.08–2.58)	0.022
Relationship status: Single	1.60 (1.12–2.29)	0.010

*95% CI: 95% confidence interval.

**Table 4:** Significant associations between the demographic characteristics and burnout using multiple logistic regression (*n* = 893).

Burnout Dimension and Demographic Characteristics *	Nagelkerke R^2^	Adjusted Odds Ratio (95% CI)	*p*-Values
Personal-related burnout	0.288		
Perceived psychosocial support received at work: Inadequate		5.50 (3.80–7.97)	<0.001
Suffering from some medical illness		2.78 (1.87–4.13)	<0.001
Child support at home: Inadequate		1.87 (1.19–2.95)	0.007
Work more than 60 h per week		1.82 (1.29–2.58)	0.001
Direct involvement with COVID-19		1.60 (1.17–2.18)	0.003
Age: Less than 40 years old		1.55 (1.05–2.27)	0.027
Work-related burnout	0.338		
Perceived psychosocial support received at work: Inadequate		5.81 (4.12–8.19)	<0.001
Work more than 60 h per week		2.65 (1.87–3.75)	<0.001
Suffering from some medical illness		2.31 (1.56–3.42)	<0.001
Spirituality routines: Irregular		1.94 (1.26–2.99)	0.003
Child support at home: Inadequate		1.92 (1.22–3.02)	0.005
Direct involvement with COVID-19		1.68 (1.22–2.32)	0.002
Patient-related burnout	0.200		
Perceived psychosocial support received at work: Inadequate		3.49 (2.38–5.13)	<0.001
Direct involvement with COVID-19		2.21 (1.50–3.26)	<0.001
Age: Less than 40 years old		1.86 (1.03–3.39)	0.041
Suffering from some medical illness		1.85 (1.20–2.86)	0.006

* Only statistically significant (*p*-values < 0.05) characteristics were shown in the table. * 95% CI: 95% confidence interval.

**Table 5:** Common self-reported causes of burnout in HCW.

Theme	Coding	Quotations
Workload	Working longer than usual hours	“I am involved in setting up the quarantine and low risk treatment centre, and I’ve been working until late. Once I reached home, I just bed down. At this stage there are many unexpected things—those big number of late night admissions, working late night... you just need to prepare for all kind of possibilities.” (P811, Dietician)
	Working with extra precaution measures	“The fatigue is constant. We need to stand for three to four hours in full PPE while handling samples.” (P119, Lab technologist)
	Affecting the team dynamic that previously functioned well	“Some staffs were reallocated to screening and emergency team, some had to undergo quarantine and the loads just keep increasing.” (P49, Assistant medical officer)
	Affecting every healthcare worker, including outside COVID-19 centers	“Even I am not in a COVID-19 treatment centre, our hospital receives more patients with other complaints. The load become unbearable with small number of manpower.” (P871, Medical intern)
	Bureaucracy matters	“There are loads of paper works to be done especially towards the end of the month. I have to sleep less to get them over and done with.” (P740, Nurse)
Uncertainty from pandemic	Unpredictability of the pandemic course	“I’m scared that the pandemic will last until next year. The leave is frozen and I can’t go back to my hometown. I just hope this feeling will not escalate to depression.” (P744, Pharmacist)
	Frequent SOP changes	“I need to brainstorm on ways to regulate the staffs, especially front liners. The constant change of SOP does affect the service. It is also tiresome to keep briefing the staffs.” (P104, Assistant medical officer)
	Frequent change of roles	“I am currently reallocated in the medical department. It is uncertain how long will I be in this department. Someday, I have to work in the screening area, someday in COVID-19 ward and other places.” (P22, Medical resident) “I feel anxious to handle Patient Under Investigation admissions in the quarantine centre. I have to deal both with my current role (in quarantine centre) and my original job scope that is sanitary water inspection and sampling. I feel this is physically and emotionally taxing.” (P813, Health inspector)
	Quarantine	“I’ve become more burnout after becoming Patient Under Investigation myself. I was quarantined after being exposed to a COVID-19 patient in clinic.” (P2, General practitioner)
	Own plan changes	“My wedding and master’s program registration needed to be postponed due to COVID-19.” (P746, Medical resident)
Challenged work–family balance	Affecting the previous work–family dynamic	“My children are taken care by my elderly parents at home. They are all bored and the house is in wreck as they are 24 h at home. I wanted to help with the chores but I came back from work completely tired.” (P874, Medical resident)
	Confinement impact	“I feel so burnt-out as I couldn’t visit my family who resides in other state.” (P99, Assistant medical officer)
	Fear of passing the virus to family members	“I find this emotionally exhaustive, I feel so stressed as I fear to be infected with this deadly virus. I feel that I have risk myself and my family to treat other patients.” (P275, Assistant medical officer)
	Financial loss	“My husband salary were cut. I let out my anger to my three year old toddler and the stress keeps building up.” (P229, Lab technologist)
Stretched workplace relationships	Superiors	“I needed to work on the same data entry six times just for a patient. You could just extract the information from the system but our new specialist insists to do it the old-fashioned way. I just think it’s not wise to do that when you are working in a hospital with full IT access.” (P866, Medical resident) “I got severe headaches and feeling frustrated as I was frequently scolded from my superiors. The superiors were also tired and let off the steam to us.” (P872, Medical intern)
	Colleagues	“I have to answer the WhatsApp on work-related even after working hours. I am always pressured by the doctors. The other technologists seem to not understand their role and gave the burden to me.” (P801, Lab technologist)
	Patients’ attitudes	“In this pandemic times, I got so tired when patients come to the hospital without the right indication. Some patients came for trivial complaints. And the interns ordered wrong films too.” (P421, Radiographer) “Some patients did not declare that they had close contacts with COVID-19 patients. And when I knew about it, I continuously feel anxious. I feel scared that I could spread the virus. I hope there will be a proper mechanism to fine patients who give wrong information, so they will be more transparent.” (P480, Radiographer)

**Table 6:** Common self-reported symptoms of burnout in HCW.

Theme	Coding	Quotations
Exhaustion	Emotional exhaustion	“It’s more of emotional exhaustion rather than physical.” (P820, Pharmacist) “Exhausted mentally, and sometimes this leads to physical exhaustion as well.” (P531, Medical intern)
	Physical exhaustion	“I’m just too tired even to think.” (P617, Lab technologist) “I feel like crawling to my car when work finishes. It feels like the hospital and my house are so far away (than it is).” (P231, Lab technologist) “I sometimes don’t drive home because I was just too tired and I slept in the on call room instead.” (P167, Specialist doctor) “Working for a day sometimes feels like a week because we are tired physically and mentally. It might appear easy but donning PPE takes time and we wear them for so long.” (P153, Lab technologist)
	Frustration	“Mentally exhausted, as we need to care for other person and their well-being, while we have our own struggle at home with these home schooling (due to COVID-19). Both of us are working, kids need to be sent to their grandparents, homework keep coming through those WhatsApp group everyday leaving us feeling even more hopeless. I cannot help but feel very guilty to the kids.” (P244, Specialist doctor)
Cynicism	Distant attitude towards work	“The first sign for me was fatigue, then feeling weary, I just go like, “Another mass sampling!”. Third, I become demotivated, numb and work just to finish them (rather than wholeheartedly). Only God understand the fatigue.” (P894, Assistant medical officer) “...I don’t think this (working in COVID-19) is worth it.” (P277, Medical resident) “I have to work from 8am to 5pm while other people can enjoy their quarantine at home.” (P191, Lab technologist) “I am easily angry over my job and there was once I nearly punch someone.” (P719, Health inspector)
	Callous comments about patients or others	“I am so resentful of those patients who seems disgusted by the front liners wearing PPE.” (P333, Paramedic) “I feel sick hearing COVID-19 word. Every patients use COVID-19 as excuses. I just feel like telling people off when they say that word.” (P742, Pharmacist)
Reduce personal accomplishment	Loss of enthusiasm or work purpose	“I’ve lost motivation to continue my usual work.” (P861, Allied health worker) “I’ve no motivation to come to work almost every morning as the annual leave is frozen. The patient load is huge and the workforce is barely adequate.” (P605, Pharmacist)
	Feeling of underperforming	“Exhausted and not productive at work.” (P540, Hospital food preparation personnel) “I feel tired and sleepy after working only half day through. I have no energy to work in the evening.” (P794, Pharmacist) “I feel numb and lack of motivation at the morning. I don’t feel prepared mentally to start work since the job scope changes during COVID-19.” (P811, Social worker)
	Low self-esteem	“I just feel giving up on everything.” (P447, Lab technologist) “I feel hopeless and useless for the past 3 weeks, life is just work.” (P18, Medical resident)

**Table 7:** Common self-reported coping strategies among burnt-out HCW.

Theme	Coding	Quotations
Problem-focused coping	Active coping	“I arranged myself for counselling session and psychiatric assessment” (P842, Specialist doctor) “I take intermittent break and do some light exercises when I feel exhausted or drenched in sweats from wearing PPE for hours to handle diagnostic tests.” (P155, Lab technologist) “Teamwork, sharing the load with your teammates, and getting the support from the management keep me going. I stayed at the accommodation centre provided by the ministry.” (P811, Dietician)
	Planning	“There’s a lot on my mind every time I wake up. My body feels heavy. Sometimes I had decision-paralysis. But I try to set up realistic aims and ‘just do it’.” (P854, Pharmacist)
Positive thinking coping	Positive reinterpretation	“Just stay strong and remind myself that the public needs me” (P85, Assistant medical officer) “...just try to think positive. Sometimes when you got stressed up, I sing or dance a bit, as long as I feel positive. I also ask for help when necessary. My team is the best.” (P153, Lab technologist)
	Acceptance	“Accept the situation wholeheartedly, put my reliance to God and keep moving forward.” (P894, Assistant medical officer) “I just come to work and return home when work is done. I have to keep going as this is what I do for a living.” (P118, Lab technologist)
	Humor	“I try to crack some jokes.” (P283, Social worker)
Seeking social support	Emotional support	“I shared my feelings with 90% of my colleagues who are also burnt out.” (P866, Medical resident) “I shared what happened during the day to my spouse.” (P197, Assistant medical officer)
	Instrumental support	“I talk about it to my colleagues, share some experience, because it is not an individual problem so we help out each other.” (P153, Lab technologist)
	Spirituality	“I keep praying to Allah that this will be over soon.” (P71, Assistant medical officer) “I recite the Quran to find some peace.” (P356, Social worker)
Maladaptive coping	Self-distraction	“I tried to find other source of happiness to forget about work stress. Those stress will be gone for a while but I still be troubled about the workload there and then.” (P519, Medical intern)
	Venting	“I JUST VENT MY ANGER AT HOME.” (P726, Medical resident) “Sometimes I accidentally take it out on patients, but if I am too frustrated or angry I just take it out on them. It might taint the image of healthcare worker but some patients are rude too.” (P533, Medical resident)
	Behavioral disengagement	“I just asked my housemen to do all (the duties).” (P476, Medical resident) “I kind of ignore the stress.” (P890, Assistant medical officer)
	Substance abuse	“I take alcohol after work (to ease the stress).” (P848, Assistant medical officer)

**Table 8:** Common self-reported burnout impacts in HCW.

Theme	Coding	Quotations
Occupational	Lack of focus	“The burnout takes away my focus at work especially when dealing with fussy patients.” (P589, Assistant medical officer) “This burnout does affect my concentration and decision making ability.” (P400, Specialist doctor) “I did not realize that I drive to a different health clinic because I was sleepy. I was working long hours till late night and needed to response to WhatsApp messages to make sure the supplies are adequate.” (P195, Pharmacist)
	Loss of enthusiasm	“I feel like not coming out from my car to work.” (P75, Allied health member) “I feel less motivated to give my best to the patients.” (P241, Medical resident) “I feel less prepared psychologically especially when starting the duties at the morning. The MCO had many sports and recreational outlets closed, when I need them to reduce the work stress.” (P811, Social worker)
	Lack of productivity	“Yes, I am not productive as I used to. I cannot continue my postgraduate revision due to exhaustion.” (P634, Medical resident) “Day by day I was not able to give the quality of work expected from me.” (P781, Assistant medical officer)
	Affecting the professional relationship with patients	“These burnout sometimes affect our communication with patients.” (P426, Paramedic)
	Affecting professionalism	“I tend to find excuses to be absent from work.” (P676, Medical resident)
	Escape fantasies	“There is not much in terms of personal life quality. I always sit down and think how do I run away from all these.” (P210, Lab technologist) “I feel like quitting and thinking about just staying at home.” (P361, Radiographer)
	Sick leave	“I have to take a lot of sick leave from physical illness resulting from burnout.” (P608, Assistant medical officer)
Physical	Headaches	“My body is exhausted, I got frequent headaches as my mind is focusing hard on the never-ending job.” (P862, Lab technologist)
	Muscular pain	“I got muscle aches at my back, shoulders and neck.” (P111, Medical resident)
	Sleep disturbance	“Every time I got home, I fall asleep easily and frequently awake at night. The sleep quality is bad.” (P203, Assistant medical officer) “In this pandemic time, it’s really hard to fall asleep and wake up at the morning to work. I had panic attacks at nights and was awake several times because of palpitation.” (P587, Pharmacist)
	Ailments	“I had frequent episodes of upper respiratory tract infection.” (P781, Assistant medical officer) “My blood pressure and heart rate went up many times.” (P174, Lab technologist)
	Palpitations	“I always get palpitations and difficulty sleeping at night. I am scared that I might have done some mistakes, I feel worthless.” (P676, Medical resident)
	Loss of appetite	“I have lost appetite, feel down and less happy.” (P640, Assistant medical officer)
	Near-/Multi-vehicle accidents	“I got so exhausted and involved with an accident. My car crashed into three other cars due to microsleep. Thank God, both of my children were unhurt.” (P761, Medical resident)
Psychological	Irritability	“I become easily angry and irritable while waiting for my own COVID-19 test result. I am worried for my family if I am infected. I seem to lose my passion in this career.” (P598, General practitioner) “I came back from work with unstable emotion and everything is not right. I lashed it out to my innocent children.” (P520, Hospital food preparation personnel)
	Anger outbursts	“I am easily enraged over small mistakes by the interns during ward round.” (P63, Specialist doctor) “I had more fights with family members and co-workers.” (P846, Medical resident)
	Anxiety symptoms	“I get chest pain episodes since COVID-19 started. It just happened when I kept thinking about work or schedules.” (P154, Lab technologist) “Every time I get sore throat, tired or runny nose, I think it is COVID-19. The anxiety is real.” (P210, Lab technologist) “I work in fear. I came to work thinking all of worst possibilities. After work, I disinfect all of my belongings just to prevent possible transmission.” (P424, Pharmacist) “I get exhausted mostly from frequent PPE changes when attending patients. I am tired mentally. I am worried that I could spread the virus that I had to distract myself from those thinking. I avoid socializing with my family because of this worry.” (P677, Medical intern)
	Extreme guilt	“Mentally exhausted, as we need to care for other person and their well-being... kids need to be sent to their grandparents... I cannot help but feel very guilty to the kids.” (P132, Specialist doctor)
	Feeling worthless	“I feel useless and aimless.” (P588, Nurse)
Social	Family relationship	“This (pandemic) made things very difficult for me and my partner.” (P601, Medical resident) “I fell asleep as soon as I reached home. I’ve lost the time with my wife and kids.” (P632, Lab technologist)
	Quality of life	“I’ve spent all my energy at work and I have barely any time to do anything else I like doing.” (P597, Nurse) “I cannot do any social activities or going back to my family during the break. I just lay down and unable to wake up even to have proper meal.” (P800, Lab technologist)
No impact	Coping well	“It didn’t affect me much. I am used to this.” (P165, Medical intern) “I started to feel those burnout symptoms towards the end of the week but I find them manageable.” (P197, Allied health member) “There is no obvious sign that it impact my work quality. We are used to on-call and overtime so that helps us to adapt to the pandemic.” (P719, Lab technologist)

## Data Availability

The data presented in this study are available on request from the corresponding author.
